# Improving prediction of bacterial sRNA regulatory targets with expression data

**DOI:** 10.1093/nargab/lqaf055

**Published:** 2025-05-08

**Authors:** Yildiz Derinkok, Haiqi Wang, Brian Tjaden

**Affiliations:** Department of Computer Science, Wellesley College, Wellesley, MA 02481, United States; Department of Computer Science, Wellesley College, Wellesley, MA 02481, United States; Department of Computer Science, Wellesley College, Wellesley, MA 02481, United States

## Abstract

Small regulatory RNAs (sRNAs) are widespread in bacteria. However, characterizing the targets of sRNA regulation in a way that scales with the increasing number of identified sRNAs has proven challenging. Computational methods offer one means for efficient characterization of sRNA targets, but the sensitivity and precision of such computational methods is limited. Here, we investigate whether publicly available expression data from RNA-seq experiments can improve the accuracy of computational prediction of sRNA regulatory targets. Using compendia of 2143 *Escherichia coli* RNA-seq samples and 177 *Salmonella* RNA-seq samples, we identify groups of co-expressed genes in each organism and incorporate this expression information into computational prediction of sRNA targets based on machine learning methods. We find that integrating expression information significantly improves the accuracy of computational results. Further, we observe that computational methods perform better when trained on smaller, higher quality sets of targets rather than on larger, noisier sets of targets identified by high-throughput methods.

## Introduction

Small regulatory RNAs (sRNAs) in bacteria are noncoding RNA genes that commonly function as post-transcriptional regulators. The vast majority of sRNAs are *trans*-acting and base pair with messenger RNA (mRNA) targets, regulating the targets’ stability or translation [1]. sRNAs are important components of regulatory networks that enable bacteria to respond rapidly, for instance, to environmental stresses [2]. While the identification of sRNA genes throughout bacterial genomes has exploded in recent years, thanks in part to the prevalence of RNA-seq studies, characterization of their targets and their functional roles has proven more challenging [3].

The most reliable methods for determining targets of a sRNA regulator are focused experimental approaches such as *lacZ* or GFP reporter assays based on compensatory mutations of a target interaction [4]. While these methods can provide high-confidence evidence of a regulatory interaction between a sRNA and one of its targets, the methods are resource intensive to conduct and relatively few interactions have been confirmed by these means. In an effort to develop methods for target detection that better scale with the rapid increase in identified sRNAs, a number of experimental methods have been developed for global target detection. One family of genome-wide approaches makes use of the fact that sRNAs and their mRNA targets often copurify with RNA-binding proteins such as Hfq, ProQ, and CsrA, at least in many Gram-negative bacteria such as *Escherichia coli* and *Salmonella enterica* [5]. Thus, approaches such as co-immunoprecipitation [6], CLIP-seq [7], and Grad-seq [8] are able to suggest candidate targets based on binding to RNA chaperone proteins. Other global approaches, such as MAPS [9], RIL-Seq [10], and GRIL-Seq [11], are able to directly capture interactions between sRNAs and all other RNAs. Some of these genome-wide approaches have compelling properties, including direct detection of interacting sRNAs and their targets, not necessarily requiring RNA-binding proteins, being effective *in vivo*, and globally identifying all RNAs (mRNAs, RNA traps, anti-sRNAs, sponges) that form transient complexes with sRNAs [12]. One of the challenges with these methods, however, is that they can detect large numbers of interactions, not all of which may be regulatory or functional in nature, e.g. in *E. coli* RIL-seq detected 1522 sRNA:target interactions [13] and CLASH detected 1733 interactions [14].

While experimental methods may be applied genome-wide for identifying sRNA targets, they remain relatively costly and cannot keep pace with the accelerated rate of detection of sRNAs throughout bacteria, particularly in the context of widespread use of RNA-seq experiments. Thus, computational methods, which are generally more efficient than experimental approaches, may better scale and can be used as a starting point for sRNA regulatory target characterization. Computational methods normally use some combination of features to predict likely targets of a sRNA, the most common of which are energy of hybridization between the two interacting RNAs [15], structural accessibility of the region of interaction within an RNA [16], the presence of a “seed” region of (∼7) consecutive base pairs in an interaction [17], and conservation of the interaction in closely related genomes [18]. Recently, machine learning methods have been used to evaluate different features and integrate the features in unified models that optimize target prediction based on existing data sets of targets of bacterial sRNA regulation [19, 20].

In this study, we consider incorporating a new set of features that have not been used previously for sRNA target prediction. The new features are based on expression data from a compendium of RNA-seq experiments. While co-expression of a sRNA and possible target does not imply regulation, and lack of co-expression does not imply there is not a functional interaction between sRNA and target, we hypothesize that co-expression and other expression patterns across a large and disparate set of expression data can provide some signal to help distinguish targets of sRNA action. We test this hypothesis by integrating information from existing repositories of bacterial expression data and evaluating improvement in target prediction. Specifically, using the machine learning and signal processing technique of independent component analysis (ICA), we construct regulatory modules from a compendium of RNA-seq data [21]. ICA is a well established and leading method for identifying groups of genes that vary consistently across conditions and for recovering regulatory networks from massive expression data sets [22]. We quantify the propensity of genes to belong to the same module as a sRNA, as determined from a compendium of expression data, and we assess the extent to which these expression-derived data enhance target identification.

After computing new expression features implicating targets of sRNAs, we interrogate the performance of different machine learning algorithms in identifying targets of a sRNA. We investigate the performance of the machine learning algorithms using previously used features as well as the new features that we propose in this study. We find that the machine learning algorithm of gradient boosting achieves the best performance and we design and train a gradient boosting model for predicting targets of sRNA regulation.

In order to test our hypotheses and evaluate our model, we employ a compendium of 2143 publicly available RNA-seq experiments from *E. coli*, since sRNAs are best understood in *E. coli* [22]. To further validate our method, we then apply it to *S. enterica* using a compendium of 177 publicly available RNA-seq experiments [23]. *Escherichia coli* and *S. enterica* provide useful contexts for evaluation of sRNA target prediction because, unlike other bacteria, they have reasonably large sets of well studied and experimentally validated sRNA regulatory interactions [4]. In the case of *E. coli*, we generate three datasets on which to assess our model. The three datasets range in size from small to large and in quality from high quality to low quality. For example, the smallest data set consists of 134 interactions that correspond to high confidence targets based on focused experimental validation, and the largest data set consists of 4158 interactions that correspond to low confidence targets based on genome-wide target identification methods. Ultimately, we find that our new expression features boost the machine learning model’s performance in predicting targets of sRNA regulation. Further, we find that a model trained on a smaller set of high quality targets performs better than a model trained on a larger set of lower confidence targets.

## Materials and methods

### Datasets

Compendia of RNA-seq datasets were downloaded from the Sequence Read Archive for *E. coli* K-12 MG1655 [22] and for *Salmonella* [23] (Supplementary Table S1). Details of the RNA-seq datasets, which reflect a broad range of experimental designs and conditions, are provided in Supplementary Table S1. For *E. coli*, sets of 134, 386, and 4158 putative sRNA:target interactions were acquired for the *small* dataset [4], the *medium* dataset [24], and the *large* dataset [19, 25], respectively. Similarly, a set of 122 putative sRNA:target interactions was acquired for *Salmonella* [4]. For each sRNA in a dataset, every mRNA in the genome that was not identified in the dataset as a regulatory target of the sRNA was considered a *noninteraction*.

### Independent component analysis

Sequencing reads were aligned to the genome using the program hisat2 version 2.2.1 (-k 1 –fast) [26] and reads mapping to genes were determined using the program featureCounts version 2.0.6 [27]. Normalized log TPM (transcripts per kilobase million) values were then calculated for each gene in each sample. Independent components were computed from the TPM values using sklearn’s FastICA package for 1000 000 iterations with 500 or 100 components for *E. coli* or *Salmonella*, respectively [28]. The number of components was a hyperparameter tuned based on model performance on validation data. For each organism, ICA resulted in a source (signal) matrix, *S*, indicating the relative membership of each gene in each component, and an activity (mixing) matrix, *A*, indicating the relative activity of each component in each sample.

### Feature values

Fifteen features were used to predict regulatory targets, nine of which were obtained from a previous study [20] and six of which are new to this study. The nine features that have been used previously include (i) the hybridization energy of a sRNA and target, which considers both the accessibility of the interacting regions and the strength of the hybridization, (ii) the distance in nucleotides between the start codon of a target and the nearest upstream gene on either strand, (iii) the distance in nucleotides between the start codon of a target and the nearest upstream gene on the same strand as the target, (iv) the number of other species that contain homologs of both the sRNA and target, (v) the length of the target coding region, (vi) whether the sRNA and target contain a seed region of seven consecutive basepairs within their putative interaction, (vii) whether the gene upstream of the target on the same strand overlaps the target start codon, (viii) whether the gene upstream of the target on either strand overlaps the target start codon, and (nine) whether there are other species that contain homologs of both the sRNA and target.

The six new expression features were determined, as follows, from the TPM values corresponding to gene expression profiles and the ICA source matrix corresponding to gene membership in each component. For the gene expression profile and ICA membership profile of each sRNA and candidate mRNA target, the distance and similarity were calculated based on the *L*^2^-norm and Pearson product–moment correlation, respectively, yielding four of the six new expression features. While the ICA source matrix provides a continuous measure of each gene’s membership in each component, we also considered binarized membership of each component. For each component, to determine which genes are binarily in the component or not, we sorted all genes in a component based on the magnitude of their membership in the component as determined by the ICA source matrix. We then repeatedly removed genes by descending magnitude as long as the remaining genes in the component passed the Kolmogorov–Smirnov normality test [29], following a previously described method [30]. Two of the six new expression features correspond to whether a sRNA and a target occur in the same ICA component or not and the strength of the target’s membership in the component as determined from the ICA source matrix.

### Machine learning methods for identifying interactions

Each of the four datasets of interactions and noninteractions (*E. coli* small, *E. coli* medium, *E. coli* large, *Salmonella*) was split into training data and testing data. To combat overfitting of properties specific to a sRNA, data were split based on sRNAs rather than based on interactions, i.e. for each sRNA, either all of its interactions and noninteractions were in the training dataset or all of its interactions and noninteractions were in the testing dataset. Values for each feature were normalized to have zero mean and unit variance with respect to the training data. Given these data, the problem of distinguishing interactions from noninteractions, i.e. targets from nontargets, is an instance of a supervised binary classification problem.

A variety of machine learning algorithms and hyperparameters were evaluated on validation data obtained from the training dataset in order to determine the algorithm and set of parameters that performed best on the validation data at identifying target interactions [31]. Machine learning algorithms that were evaluated include logistic regression, neural networks, gradient boosting, random forests, *k*-nearest neighbors, and support vector machines. Details of the performance of each algorithm with different hyperparameter values are provided in Supplementary Table S2. Because the data are highly imbalanced with ∼99% of candidate interactions corresponding to noninteractions and ∼1% of candidate interactions corresponding to interactions, we evaluated randomly undersampling the majority class as well as oversampling the minority class via the synthetic minority oversampling technique [32]. Ultimately, we found the best performing algorithm to be gradient boosting, corresponding to an ensemble of boosted decision trees, after undersampling the majority class. Once the optimal algorithm and associated parameters were determined based on validation performance, the fitted gradient boosting machine learning model was used to make predictions for the previously unseen testing data, and all reported results correspond to performance on testing data.

## Results

### Compendium of expression data

**Figure 1.**
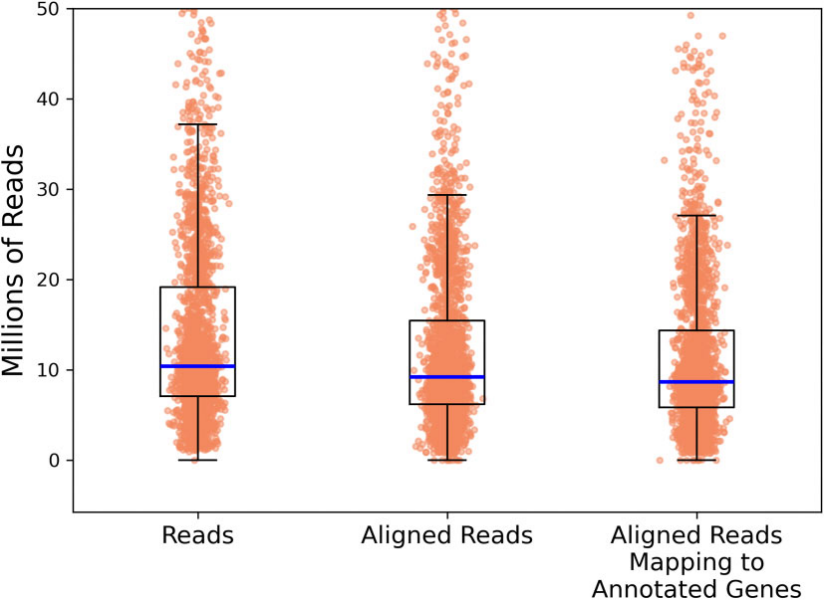
For 2143 *E. coli* RNA-seq samples, the figure shows the distribution of the number of reads, the number of reads aligning to the genome, and the number of aligning reads that map to an annotated gene in the genome. For the overlayed box and whisker plots, the horizontal line indicates the median of the distribution, the top and bottom of the box indicate the first and third quartiles of the distribution, and the whiskers extend from the box by 1.5 times the inter-quartile range.

As gene expression data have not previously been incorporated into computational tools for predicting targets of sRNA regulation, we explored to what extent existing RNA-seq data can improve target predictions. We downloaded data for 2143 *E. coli* K-12 MG1655 RNA-seq samples (Supplementary Table S1) from the Sequence Read Archive [33]. RNA-seq datasets were downloaded as part of an automated process without regard for experimental design or for assayed conditions. Altogether, the data contain >31 billion sequencing reads consisting of 4.2 trillion sequenced bases. We found that 90% of the reads aligned to the genome and, of those reads aligning, 94% mapped to annotated *E. coli* genes with the remaining 6% mapping to unannotated regions of the genome (Fig. 1).

### Groups of co-expressed genes

Based on this large expression dataset, we grouped genes into co-expression modules using the unsupervised machine learning technique of ICA [21]. ICA enables identification of statistically independent signals that modulate the expression of specific gene sets [30]. Previously, ICA has been applied to compendia of RNA-seq datasets from a broad range of organisms, including *E. coli*, owing to its effectiveness in identifying independent groups of genes whose expression varies consistently across samples [22]. One advantage of ICA over clustering is that, with ICA, genes fundamentally can belong to different ICA components, reflective of how genes can belong to different modulons and regulatory pathways. To assess the components identified by our ICA analysis, we tested whether the components differed from the null hypothesis of a random grouping, assuming a hypergeometric distribution, and observed a *P*-value for the ICA components of 8.0e-18, suggesting that the ICA components are statistically significant. To understand if components might capture aspects of a known regulon, we considered the well studied PhoP/PhoQ two-component system, which responds to a variety of environmental conditions, including low magnesium, presence of antimicrobial peptides, and acidic pH [34]. Supplementary Figure S1 illustrates a component identified by ICA that has some overlap with the PhoP/PhoQ regulon, with 13 of the 40 genes in the component being known members of the regulon.

**Figure 2.**
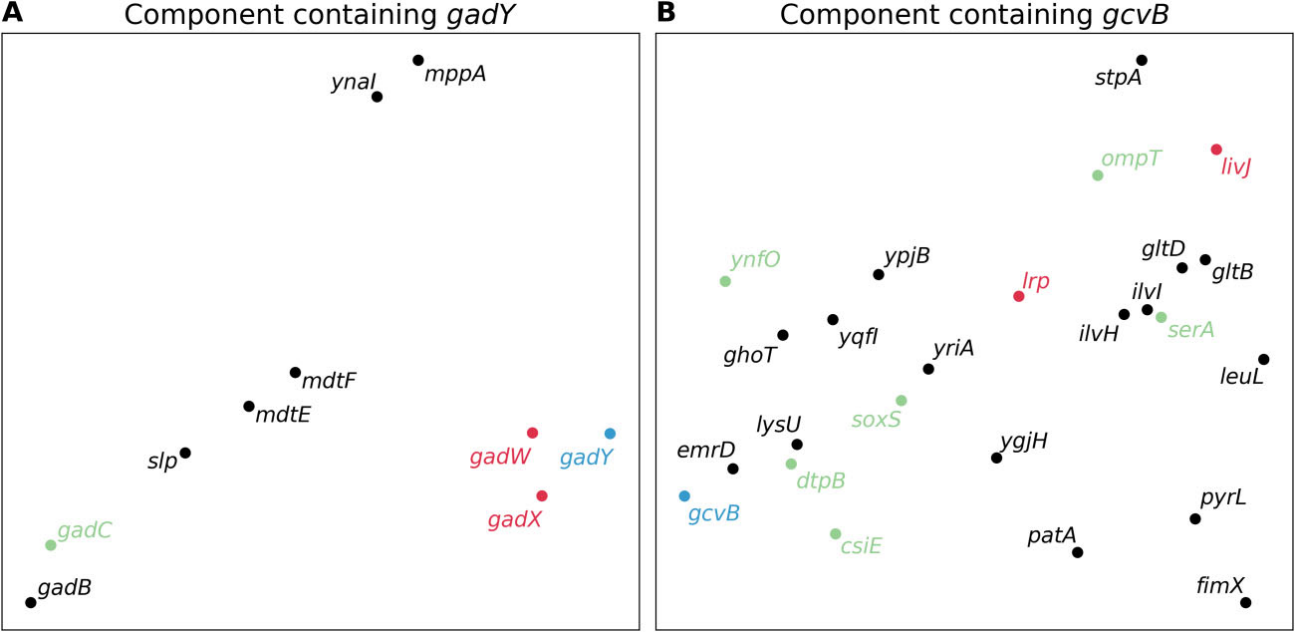
The genes in two of the components determined by ICA are illustrated. (**A**) A component consisting of 10 genes including the sRNA GadY. (**B**) A component consisting of 25 genes including the sRNA GcvB. sRNAs are shown in blue, confirmed targets of the sRNA are shown in red, and targets suggested by high-throughput approaches are shown in green. The locations of the points were determined by applying t-SNE (t-distributed stochastic neighbor embedding) to the high-dimensional expression profiles of the genes in 2143-dimensional space in order to nonlinearly embed each point in two-dimensional space for the sake of visualization. While the axes are not interpretable, the relative distances between points in the two-dimensional figure approximate the distances between points in the native 2143-dimensional space, so that points appearing closer together in the figure have more similar gene expression profiles.

We also explored modules containing sRNAs. Figure 2 illustrates two example components identified by our ICA analysis, one containing the sRNA GadY and one containing the sRNA GcvB, indicating that sRNAs and their targets may be grouped into the same component as a result of co-varying expression across RNA-seq experiments. It is important to note that components reflect shared expression patterns across a range of experiments and not necessarily direct regulation. For example, in the case of the component containing the sRNA GadY (Fig. 2A), GadY interacts with the mRNA encoding the GadX transcriptional activator, stabilizing the mRNA and increasing levels of GadX, which in turn increase the levels of the GadB glutamate decarboxylase [35]. Thus, GadY directly regulates GadX, but not GadB, though all have shared expression patterns and appear in the same component. Some other members of the component in Fig. 2A, such as *ynaI* and *mppA*, may be co-expressed with GadY under stress conditions but, likewise, are not regulatory targets.

### New features for distinguishing sRNA regulatory targets from nontargets

**Figure 3.**
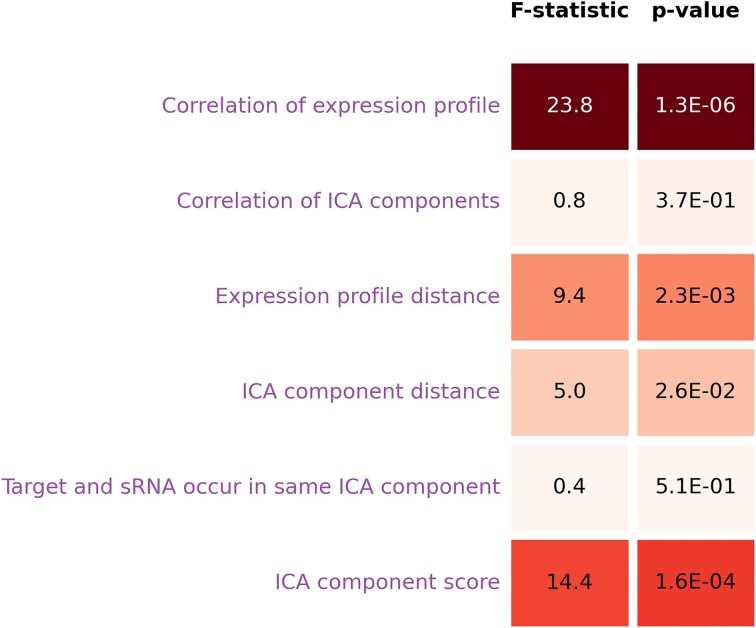
For each of six expression features, the degree of linear dependency is determined between the feature and whether a gene is a sRNA target or not. Linear dependency is estimated using an ANOVA F-test with the figure indicating the resulting F-statistic and corresponding *P*-value.

For a given sRNA regulator, we hypothesized that a known target was more likely to have an expression profile related to that of the sRNA than a gene not known to be a regulatory target. To test this hypothesis, we computed six feature values for every possible sRNA regulatory interaction and noninteraction (see the ‘Materials and methods’ section). The six features represent six different similarity (or distance) measures between the ICA expression profile of a sRNA and the ICA expression profile of a candidate regulatory target. For each of these six expression features, we calculated the ANOVA (analysis of variance) F-statistic and corresponding *P*-value indicating the dependency between the feature and whether the candidate interaction is indeed known to be a regulatory interaction or not. As shown in Fig. 3, some of the six features (e.g. whether a target and a sRNA occur in the same ICA component) have a weak relationship to whether there is a known regulatory interaction and others of the six features (e.g. correlation of expression profiles) have a strong relationship to whether there is a known regulatory interaction. Based on these results, we concluded that our newly computed expression features could help discern targets from nontargets and, if incorporated into target prediction methods, might improve the performance of such methods.

### Improving target prediction

We investigated three different datasets describing sRNA regulatory interactions in *E. coli*. The first dataset labeled *small* contains relatively few interactions (134 sRNA:target interactions and 120 404 noninteractions) but it is of the highest quality, e.g. each interaction has been confirmed by focused experiment and curated by an expert in the field [4]. The second dataset labeled *medium* contains somewhat more interactions (386 sRNA:target interactions and 189 034 noninteractions) with a combination of experimentally confirmed and computationally predicted interactions [24]. The third dataset labeled *large* contains an order of magnitude more interactions (4158 sRNA:target interactions and 249 837 noninteractions) taken from multiple databases with most interactions being identified by genome-wide, high-throughput methods or computationally predicted [19, 25].

**Figure 4.**
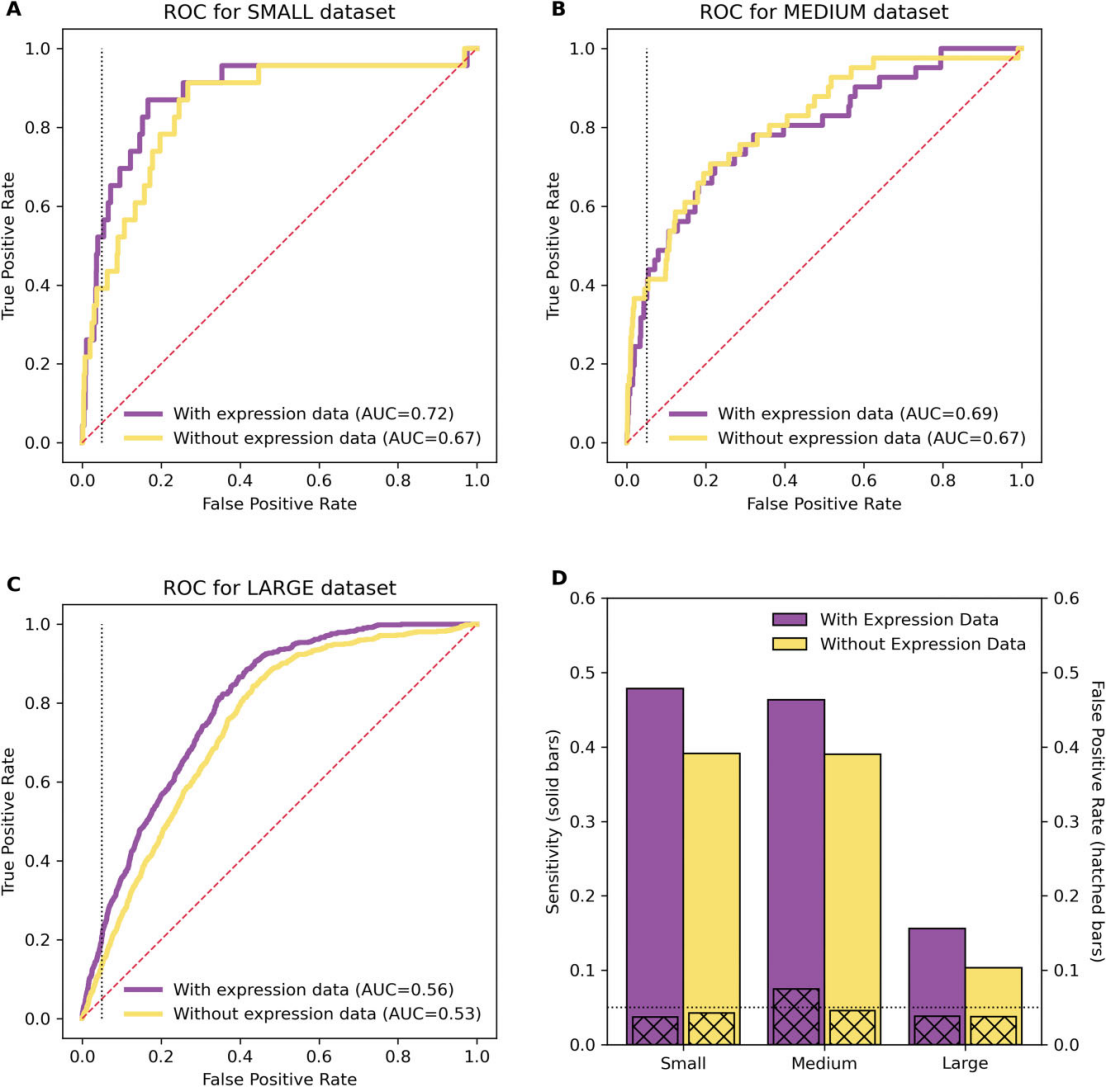
Receiver operating characteristic (ROC) curves are shown for the (**A**) small, (**B**) medium, and (**C**) large datasets from *E. coli*. The ROC curves indicate the performance of the machine learning model by showing the tradeoff between true positive rate (sensitivity) and false positive rate at different prediction thresholds. Curves closer to the top left corner indicate better performance. The dashed diagonal line indicates the performance of a naïve, random model. The dotted vertical line corresponds to a false positive rate of 0.05. The area under the curve (AUC) is also indicated. (**D**) The sensitivity (solid bars) and false positive rate (hatched bars) are shown for the small, medium, and large datasets at the default prediction threshold of 0.5 [one particular point along the curves in panels (A), (B), and (C)]. The dotted horizontal line corresponds to a false positive rate of 0.05. For all subfigures, results in yellow demonstrate performance using 9 features, not including 6 new expression features, and results in purple demonstrate performance using 15 features, including 6 new expression features.

For every interaction and noninteraction, we calculated 15 values: 9 values corresponding to features that have been used previously for predicting sRNA target interactions [20] and 6 values corresponding to new expression features developed in this study as part of our ICA analysis. We explored a variety of machine learning algorithms and their associated hyperparameters for distinguishing sRNA regulatory interactions in the three datasets, ultimately observing a gradient boosting decision tree ensemble model as the best performing (see the ‘Materials and methods’ section and Supplementary Table S2). For each of the small, medium, and large datasets, Fig. 4 shows the performance of the machine learning model using the 9 previously used features and using 15 features including our 6 new expression features. As illustrated in Fig. 4, sRNA target prediction is most accurate when the model is trained on the small, high-quality dataset (AUC of 0.72, sensitivity of 0.48, false positive rate of 0.04). With the medium and large datasets, which contain increasingly more interactions identified by computational predictions or by high-throughput methods rather than by focused experiments, the performance degrades (AUCs of 0.69 and 0.56, sensitivities of 0.46 and 0.16, false positive rates of 0.07 and 0.04, respectively). Importantly, in all cases, incorporating the six new expression features improves the performance of the machine learning model, as measured by AUC, suggesting that these expression features add value to computational identification of sRNA regulatory targets.

While ROC curves (Fig. 4A–C) demonstrate a variety of sensitivities and false positive rates, we are especially interested in low false positive rates since, in many applications, generating a large number of spurious target predictions for a sRNA (a high false positive rate) has limited utility. Thus, we focus our attention on low false positive rates (the dotted black lines in Fig. 4 correspond to a false positive rate of 0.05). The default prediction threshold for our model is 0.5, i.e. if it determines that a sRNA and target interact with probability greater than or equal to 50% then the models predicts an interaction, whereas if it determines that a sRNA and target interact with probability <50% then the model predicts there is not an interaction. Changing the prediction threshold from 0.5 leads to different points along the ROC curve. Figure 4D shows the sensitivity and false positive rate using the default threshold of 50%. A model trained on small, high-quality data using the new expression features achieves a sensitivity of 48%, i.e. the model is expected to accurately identify 48% of new interactions, with a false positive rate of 4% (Fig. 4D). The model’s prediction for each sRNA and each candidate target is provided in Supplementary Table S3.

Despite our emphasis on low false positive rates, our approach, similar to other tools [20], may still make dozens or in some cases even hundreds of target predictions for a given sRNA, which is often significantly more than the number of known targets for the sRNA. We consider all predictions that do not correspond to a known target as false positives, though some may indeed be as yet to be characterized targets and, therefore, true positives rather than false positives. Nevertheless, the high ratio of false positive predictions to true positive predictions results in a lack of precision. Supplementary Figure S2 shows a precision recall curve, which illustrates the tradeoff between precision and recall (sensitivity). While the precision is higher when using 15 features, including our 6 new expression features (average precision score of 0.043), compared to using just 9 features (average precision score of 0.027) or a random model (average precision score of 0.013), there remains significant room for improvement in the precision of our approach (Supplementary Fig. S2).

A relatively large number of RNA-seq datasets are publicly available for *E. coli* in comparison to many other bacteria. Thus, we wanted to explore how the performance of our model changes when fewer RNA-seq datasets are used. We took random subsets of the 2143 *E. coli* RNA-seq datasets and trained a model on data from each subset. Supplementary Figure S3 shows the sensitivity of models trained on data from different random subsets of the 2143 *E. coli* RNA-seq experiments. In general, models performed better when trained on data from larger sets of RNA-seq experiments. As illustrated in Supplementary Fig. S3, the performance can be reasonably approximated by a line with a small positive slope. While more data leads to better performance up to 2143 RNA-seq experiments, we cannot say if this upward trend would continue with additional experiments beyond 2143 or perhaps plateau at some point.

### Contribution of different features

**Figure 5.**
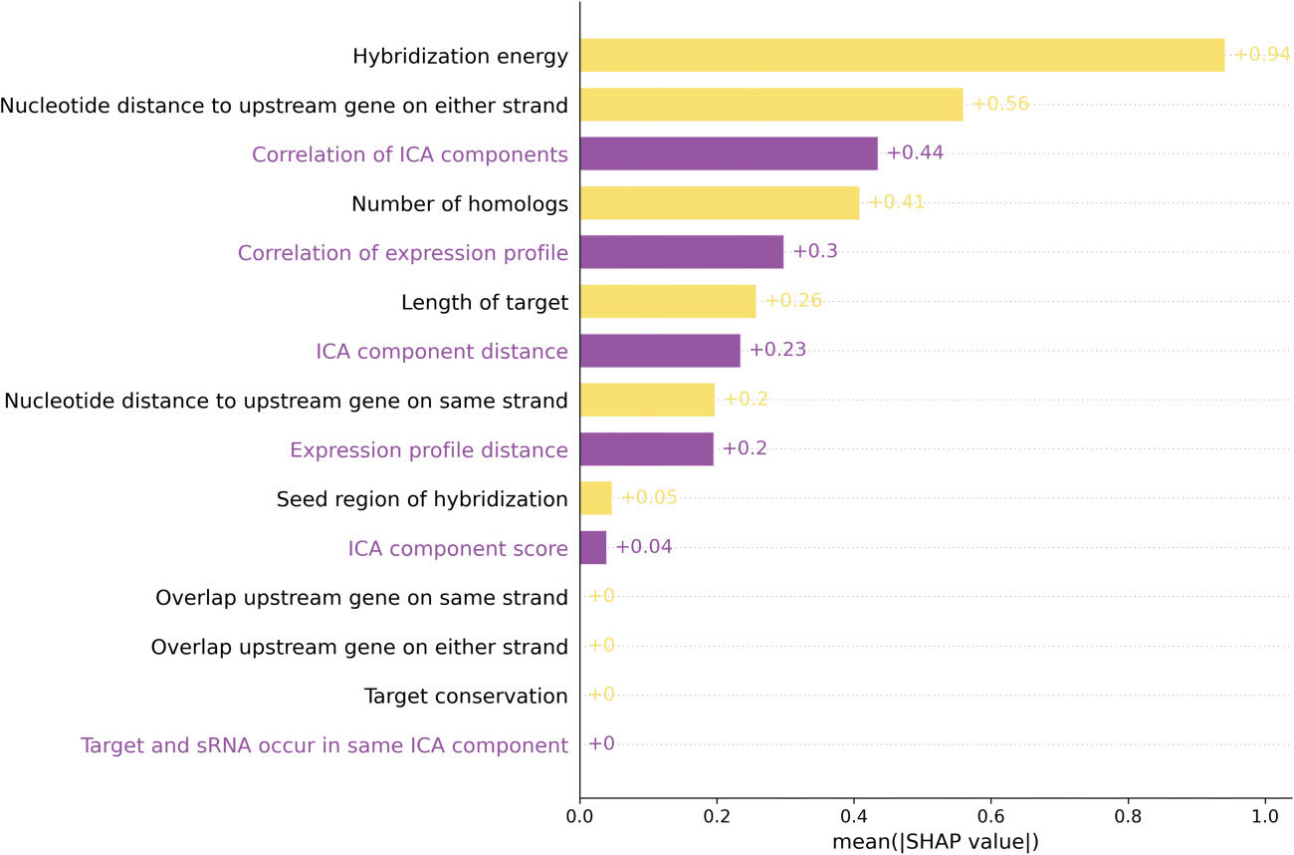
Shapley values are indicated for the 15 features, with the 6 new expression features indicated in purple. Larger Shapley values for a feature correspond to a greater contribution toward the model’s output.

To investigate the explainability of our machine learning model toward distinguishing interactions from noninteractions, we computed Shapley values for each of the 15 features. Shapley values correspond to the average marginal contribution of each feature toward the model’s output [36]. Figure 5 shows the contribution of each feature in the model, as determined by Shapley value, toward identifying interactions based on the small, high quality dataset. As shown by the values in Fig. 5, we find that many of the new expression features (in purple in the figure) make significant contributions to the machine learning model’s performance. The new expression features do not correspond, for example, to the two most impactful features, so features that have been used previously for target prediction should continue to be used, but the new features generally add meaningful value to the predictive power of the model beyond the contributions of previously employed features.

It is worth mentioning that some of the nine previously used features may have helped lead to the discovery of a number of sRNA:target interactions that we are using to evaluate the model and compute Shapley values. Thus, it is possible that there is some bias in our dataset that leads to higher Shapley values for the nine previously used features. It is also important to note that the features are not independent. For instance, of the six new features, the two that contribute least toward the model’s output are “ICA component score” and “Target and sRNA occur in same ICA component”, the former being a decimal number indicating the level of a target’s membership in the same component as a sRNA and the latter being a binarized (Boolean) version of the former where the target is considered as being in the same component as a sRNA or not. Based on Shapley values (Fig. 5), these two features appear to contribute little beyond what the other features are capturing. Features with minimal contribution could reasonably be removed, though there is little advantage in doing so, since they are not detracting from the model’s performance but possibly enhancing slightly the model’s robustness.

### Comparison with RIL-seq data

RIL-seq is a leading experimental approach for global identification of sRNA:target interactions [13]. We examined how predictions from our model compare to target interactions identified by RIL-seq experiments. Supplementary Figure S4 shows, for the 134 confirmed interactions in the small *E. coli* dataset [4], how many were identified by RIL-seq and how many were predicted by our model. Overall, 51% of the interactions were identified by RIL-seq and 80% were predicted by our model. It is important to note that these results include both training and testing data. On testing data alone, which is a more reasonable indicator of future performance on new data, our model correctly predicted 48% of the interactions (Fig. 4D). We also delved into GcvB, since this sRNA has one of the largest numbers of characterized targets. RIL-seq experiments suggested 121 targets for GcvB, and of these 121 suggested targets, our model predicted 58% of them (Supplementary Fig. S5).

### Predicting sRNA targets in *Salmonella*

**Figure 6.**
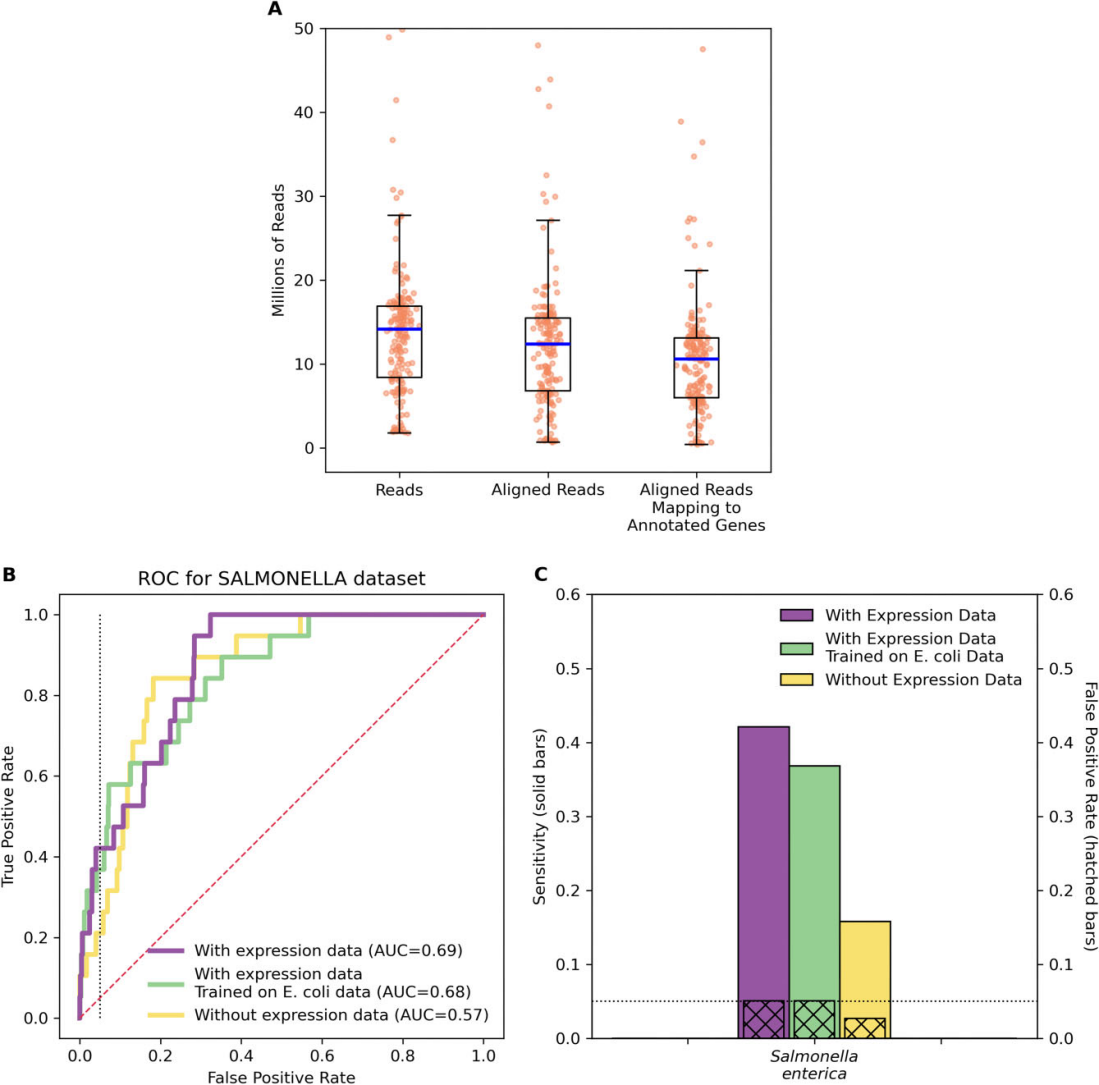
(**A**) For 177 *Salmonella* RNA-seq samples, the figure shows the distribution of the number of reads, the number of reads aligning to the genome, and the number of aligning reads that map to an annotated gene in the genome. For the overlayed box and whisker plots, the horizontal line indicates the median of the distribution, the top and bottom of the box indicate the first and third quartiles of the distribution, and the whiskers extend from the box by 1.5 times the inter-quartile range. (**B**) ROC curves are shown for the *Salmonella* dataset indicating the performance of three machine learning models by showing the tradeoff between true positive rate (sensitivity) and false positive rate at different prediction thresholds. One model was trained on *Salmonella* data using expression features, one model was trained on *E. coli* data using expression features, and one model was trained on *Salmonella* data not using expression features. The dashed diagonal line indicates the performance of a naïve, random model. The dotted vertical line corresponds to a false positive rate of 0.05. The AUC is also indicated. (**C**) The sensitivity (solid bars) and false positive rate (hatched bars) are shown for the *Salmonella* dataset at the default prediction threshold of 0.5 [one particular point along the curves in panel (B)]. The dotted horizontal line corresponds to a false positive rate of 0.05. For panels (B) and (C), results in yellow demonstrate performance of a model trained on *Salmonella* data using 9 features, not including 6 new expression features; results in purple demonstrate performance of a model trained on *Salmonella* data using 15 features, including 6 new expression features; and results in green demonstrate performance of a model trained on *E. coli* data using 15 features.

In order to investigate our approach in another organism beyond *E. coli*, we repeated our experimental methods using data from *Salmonella*. While numerous sRNA:target interactions have been predicted in other bacteria, e.g. via computational methods or high-throughput sequencing approaches, *Salmonella* is the only example beyond *E. coli* with a large number (>100) of rigorously studied and confirmed interactions—a large enough number to meaningfully train a machine learning model. Similar to the *small* high-quality *E. coli* set of interactions, we gathered a set of 122 sRNA:target interactions in *Salmonella* that have been confirmed by focused experimentation [4]. We then downloaded data for 177 *Salmonella* RNA-seq samples from the Sequence Read Archive (Supplementary Table S1). The samples contain 2.5 billion sequencing reads consisting of >300 billion sequenced bases, with 87% of the reads aligning to the genome, and 85% of the aligned reads mapping to annotated *Salmonella* genes (Fig. 6A). We determined groups of co-expressed genes using ICA and calculated 15 feature values for each of the 122 sRNA:target interactions and 162 058 noninteractions. We trained three machine learning models, one trained on *Salmonella* data using 15 features, one trained on *E. coli* data using 15 features, and one trained on *Salmonella* data using 9 features. For the two models trained on *Salmonella* data, we observe that incorporating the six new expression features improves our ability to discern targets from nontargets, with our model achieving a sensitivity of 42% when identifying *Salmonella* interactions while maintaining a false positive rate of no >5% (Fig. 6B and C). The model trained on *E. coli* data but evaluated on *Salmonella* data provides a glimpse into how a model trained on data from one species might perform when applied to data from another species. In this case, the model achieved a sensitivity of 37% with a 5% false positive rate.

We also explored the contribution of features in the model by computing Shapley values for each feature (Supplementary Fig. S6). The Shapley values for the model trained on *Salmonella* data (Supplementary Fig. S6) are roughly similar to the values for the model trained on *E. coli* data (Fig. 5), though some of the features have increased or decreased contributions. As with the Shapley values from the *E. coli* data, the six new expression features introduced in this study are not the most impactful features, though some but not all make meaningful contributions to the model’s output. For this model trained on *Salmonella* data, the model’s prediction for each sRNA and each candidate target is provided in Supplementary Table S3.

## Discussion

sRNA regulators are increasingly being identified across bacteria. For the best studied genomes, more than a hundred sRNA genes have been characterized throughout the genome with many more hypothesized to exist. It remains a major challenge to identify the regulatory targets of these sRNAs. Focused experimental approaches provide the best evidence for regulatory interactions between a sRNA and a target; however, these approaches do not necessarily scale with the large and growing number of characterized sRNAs. Global experimental approaches, such as RIL-seq, enable broader indication of potential targets for a sRNA, but these approaches are challenged to distinguish regulatory interactions from nonfunctional interactions between RNAs. Computational methods for predicting targets of sRNA regulation are the most efficient, but historically these methods have suffered from limited sensitivity and high false positive rates. In this study, we investigate improving computational methods by leveraging existing databases of RNA-seq datasets.

We hypothesized that gene expression information from large sets of RNA-seq experiments can help improve the performance of computational methods for sRNA target identification. To test this hypothesis, we focused on *E. coli* and *Salmonella* where large sets of sRNA regulatory interactions have been characterized previously. For each organism, we downloaded a compendium of RNA-seq datasets and extracted gene expression information across the rich set of RNA-seq samples. Rather than hand-select data from more relevant RNA-seq experiments, we used an automated process to download data from the entire distribution of available RNA-seq datasets. While a curated selection of RNA-seq data might lead to better target prediction, our aim was to evaluate an automated process free of human curation that can be applied in a scalable manner to any bacteria with a sufficient distribution of publicly available RNA-seq datasets. After obtaining this wealth of expression data, using the machine learning approach of ICA, we identified modules of co-expressed genes and investigated whether this expression information could be used to enhance prediction of sRNA regulatory targets.

In order to computationally distinguish targets of sRNA regulation from nontargets, we trained machine learning models on different sets of interactions ranging in size and quality. The models performed best when trained on high quality interactions that were identified by focused experimental methods, even though the number of such interactions was small. Conversely, when trained on larger sets of lower confidence interactions that were identified by high-throughput rather than focused methods, the performance of the models decreased. This is an interesting finding because, in general, machine learning models perform better when trained on larger datasets. However, when the increase in the size of the training dataset comes at the cost of quality, the impact on model performance is unclear and depends on context specific factors. Here, for predicting sRNA regulatory targets, we observe that computational methods perform best when based on smaller sets of confirmed data rather than on larger sets of data based on global methods that may or may not capture functional regulation. It remains an open question as to whether these findings result from noise in the high throughput methods such as high false positive rates or from the machine learning models being better able to discern patterns common to sRNA regulatory targets from the high-quality, confirmed data.

In all cases, regardless of the organism or the size and quality of the dataset, we found that incorporating expression information improved the performance of the computational methods. When integrating expression features based on large sets of RNA-seq experiments, the sensitivity of the computational methods improves, e.g. from 39% to 48% for high-quality *E. coli* data and from 16% to 42% for high-quality *Salmonella* data. This improvement enables the computational method to identify a substantially larger number of sRNA regulatory targets. Importantly, the enhanced sensitivity does not come at the cost of lower specificity, as the computational methods maintained a low false positive rate. When training a model on *E. coli* data and evaluating it on *Salmonella* data, we found a sensitivity of 37%, which is less than that for a model trained on *Salmonella* data with the same set of features (42%) but greater than that for a model trained on *Salmonella* data without expression features (16%). While this limited finding suggests there may be value in applying a model trained on data from one species to other species, we urge caution in extrapolating in this manner since there is considerable conservation among sRNAs found in *E. coli* and *Salmonella*, and this conservation may not be present among sRNAs in other species. Going forward, as large numbers of interactions are identified in other more diverse species, we will be better able to assess to what extent our results have broader applicability.

Expression data have not been used previously as part of general computational methods for predicting targets of sRNA regulation. Our findings suggest that these publicly available data should be used. Computational prediction of sRNA targets is a difficult problem. By incorporating expression data, the performance of the method can be enhanced so that sRNA targets are identified more accurately. We also find that methods are better able to distinguish sRNA:target interactions from noninteractions when based on experimentally confirmed interactions rather than on interactions suggested by high-throughput approaches. Together, these findings advance our ability to identify sRNA targets and help elucidate regulatory networks in bacteria.

## Supplementary Material

lqaf055_Supplemental_Files

## Data Availability

Data files and code are available at https://doi.org/10.5281/zenodo.14640275. Source code is available at https://github.com/btjaden/TargetExpression.
