# Supplementary material for: Improving prediction of bacterial sRNA regulatory targets with expression data
Source: NAR Genom Bioinform. 2025 May 8;7(2):lqaf055. doi: 10.1093/nargab/lqaf055 (PMC12060007; doi:10.1093/nargab/lqaf055)
Supplement: lqaf055_Supplemental_Files [file lqaf055_supplemental_files.zip › Supplementary_Material.pdf]

Supplementary Material

Improving prediction of bacterial sRNA regulatory targets with expression data

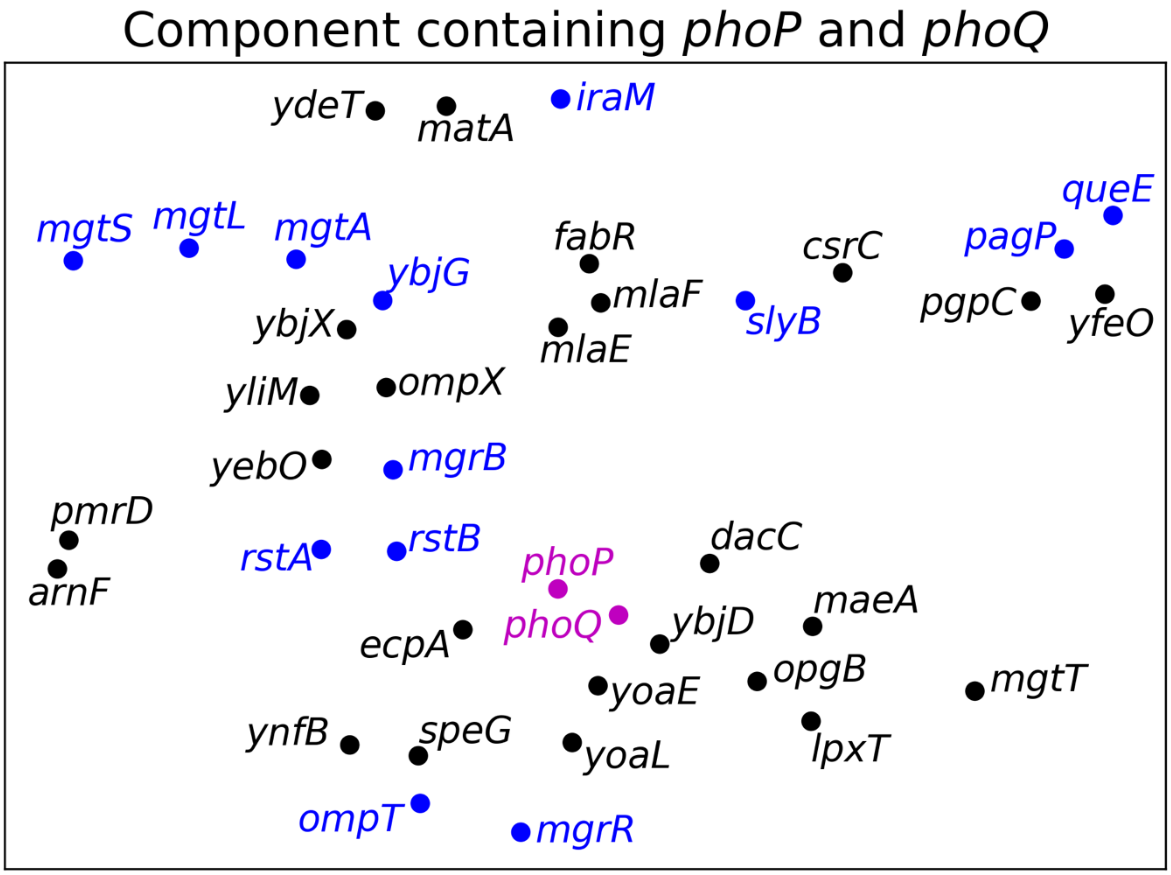

Supplementary Figure 1

A component consisting of 40 genes identified by ICA. The component includes *phoP* and *phoQ*, shown in purple, and 13 genes that are part of the PhoP/PhoQ regulon [1, 2], shown in blue. The locations of the points were determined by applying t-SNE (t-distributed stochastic neighbor embedding) to the high-dimensional expression profiles of the genes in 2,143-dimensional space in order to non-linearly embed each point in 2-dimensional space for the sake of visualization. While the axes are not interpretable, the relative distances between points in the 2-dimensional figure approximate the distances between points in the native 2,143-dimensional space, so that points appearing closer together in the figure have more similar gene expression profiles.

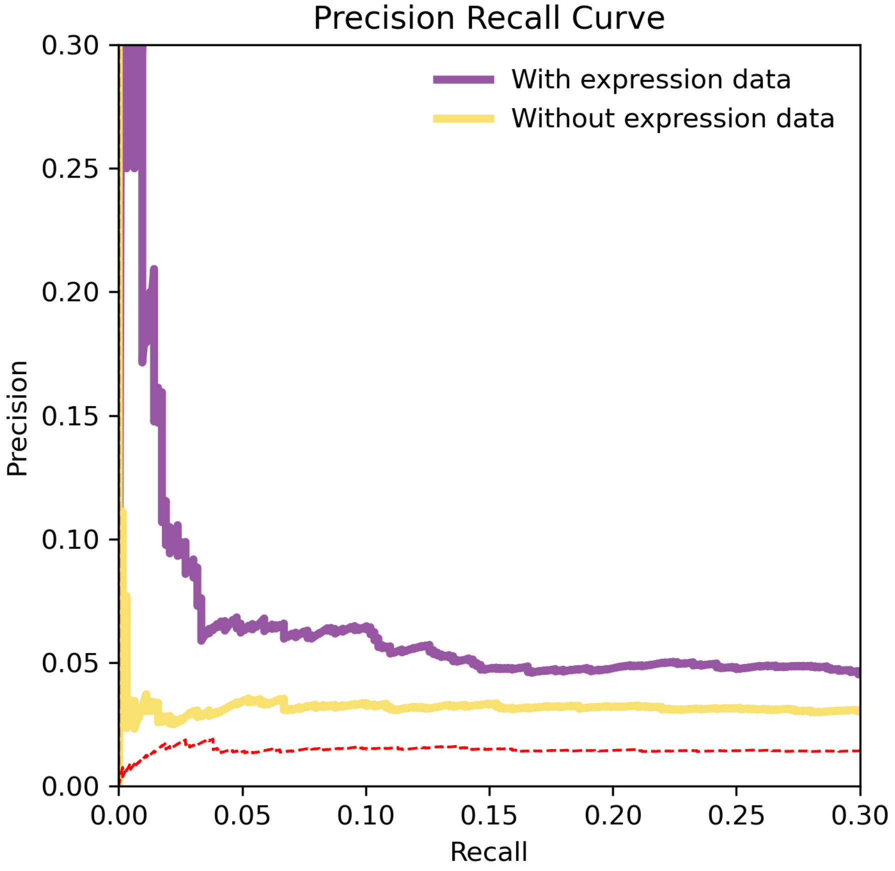

Supplementary Figure 2

Precision recall curves are shown for the large dataset from *E. coli*. The precision recall curves indicate the performance of the machine learning model by showing the tradeoff between precision and recall (sensitivity) at different prediction thresholds. Results in yellow demonstrate performance using 9 features, not including 6 new expression features, and results in purple demonstrate performance using 15 features, including 6 new expression features. The dashed red line indicates the performance of a random model.

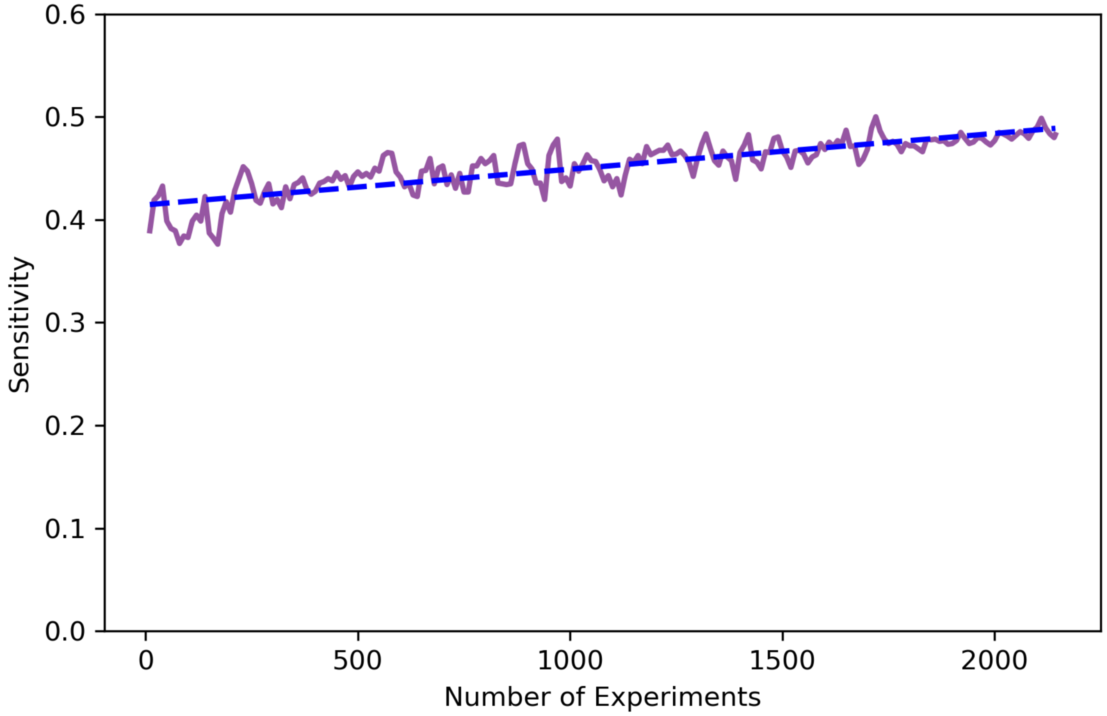

Supplementary Figure 3

The sensitivity of a machine learning model is shown when trained on expression data from different numbers of RNA-seq experiments. Each point along the purple curve corresponds to the sensitivity of a model trained on data from a random subset of the 2,143 *E. coli* RNA-seq experiments. A line of best fit is indicated by the dashed blue line.

sRNA : target	RIL-Seq (Odds Ratio)	Prediction (Probability)
ArcZ : *rpoS*	2.2	0.48
ArcZ : *arcB*	0.0	0.96
ArcZ : *flhD*	6.1	0.68
ChiX : *chbC*	0.0	0.76
ChiX : *dpiB*	0.0	0.84
ChiX : *chiP*	8.0	0.46
CyaR : *luxS*	0.2	0.42
CyaR : *nadE*	9.1	0.47
CyaR : *ompX*	6.2	0.73
CyaR : *yqaE*	1.2	0.57
CyaR : *ptsI*	0.0	0.78
CyaR : *yobF*	0.5	0.82
CyaR : *sdhA*	0.0	0.45
GcvB : *sstT*	7.1	0.98
GcvB : *cycA*	6.0	0.98
GcvB : *csgD*	0.0	0.93
GcvB : *glmS*	0.0	0.81
GcvB : *argT*	1.7	0.98
GcvB : *dppA*	5.2	0.97
GcvB : *gltI*	6.0	0.97
GcvB : *livK*	10.1	0.97
GcvB : *livJ*	11.0	0.98
GcvB : *lrp*	0.6	0.98
GcvB : *phoP*	1.0	0.58
GcvB : *oppA*	4.9	0.93
MicA : *phoP*	3.9	0.45
MicA : *ompX*	0.8	0.91
MicA : *tsx*	5.5	0.62
MicA : *ompA*	9.2	0.96
MicC : *ompC*	0.0	0.92
MicF : *lrp*	1.2	0.96
MicF : *cpxR*	0.0	0.66
MicF : *phoE*	0.0	0.84
MicF : *ompF*	23.5	0.95
OmrA : *csgD*	6.8	0.97
OmrA : *cirA*	0.0	0.87
OmrA : *ompT*	0.0	0.88
OmrA : *ompR*	0.0	0.94
OmrA : *fecA*	0.0	0.56
OmrA : *fepA*	0.0	0.85
OmrA : *flhD*	0.0	0.83
OmrA : *gntP*	0.0	0.78
OmrB : *csgD*	26.6	0.94
OmrB : *cirA*	0.0	0.9
OmrB : *ompT*	2.7	0.75
OmrB : *ompR*	2.3	0.84
OmrB : *fecA*	1.9	0.47
OmrB : *fepA*	0.0	0.74
OmrB : *flhD*	0.0	0.64
OmrB : *gntP*	78.0	0.82
OxyS : *fhlA*	0.0	0.73
OxyS : *flhD*	0.0	0.78
OxyS : *rpoS*	0.0	0.55
RprA : *rpoS*	71.1	0.85
RprA : *dgcM*	0.0	0.69
RprA : *csgD*	470.4	0.96
RybB : *fiu*	2.4	0.78
RybB : *sdhC*	0.0	0.87
RybB : *ompC*	2.2	0.62
RybB : *ompW*	13.0	0.93
RybB : *rluD*	0.0	0.57
RybB : *mraZ*	0.0	0.75
RyhB : *cysE*	1.1	0.61
RyhB : *iscS*	0.0	0.44
RyhB : *fur*	0.0	0.28
RyhB : *shiA*	46.8	0.92
RyhB : *sodB*	2.8	0.88
RyhB : *sdhC*	30.7	0.94
RyhB : *fumA*	4.8	0.81
RyhB : *nirB*	3.4	0.88
RyhB : *erpA*	4.3	0.77
RyhB : *marA*	0.0	0.46
RyhB : *nagZ*	0.0	0.9
RyhB : *sdhA*	0.0	0.93
RyhB : *acnA*	0.0	0.82
RyhB : *acnB*	1.1	0.9
RyhB : *bfr*	0.0	0.49
RyhB : *iscR*	0.0	0.64
SgrS : *ptsG*	183.4	0.98
SgrS : *manX*	18.1	0.78
SgrS : *ptsI*	0.0	0.94
Spot42 : *galK*	0.4	0.88
Spot42 : *gltA*	0.5	0.68
Spot42 : *nanC*	11.1	0.95
Spot42 : *srlA*	2.5	0.72
Spot42 : *sthA*	0.8	0.78
Spot42 : *xylF*	0.0	0.9
Spot42 : *fucI*	0.0	0.88
Spot42 : *sdhC*	0.6	0.94
Spot42 : *gdhA*	0.0	0.73
Spot42 : *sucC*	0.0	0.91
Spot42 : *icd*	0.0	0.69
Spot42 : *araF*	0.0	0.87
Spot42 : *atoD*	0.0	0.62
Spot42 : *caiA*	0.0	0.44
Spot42 : *maeA*	0.0	0.67
Spot42 : *nanT*	0.0	0.88
Spot42 : *paaK*	7.8	0.73
Spot42 : *puuE*	0.0	0.88
Spot42 : *fucP*	0.0	0.58
GadY : *gadX*	0.0	0.96
GadY : *gadW*	0.0	0.94
GlmZ : *glmS*	0.0	0.8
MgrR : *pitA*	19.4	0.69
MgrR : *soxS*	5.1	0.56
MicL : *lpp*	0.9	0.53
RseX : *ompA*	18.3	0.44
RseX : *ompC*	0.0	0.86
RydC : *csgD*	0.0	0.49
RydC : *yejA*	0.0	0.61
SdsN : *nfsA*	0.0	0.17
McaS : *csgD*	184.7	0.85
McaS : *flhD*	6.2	0.07
McaS : *pgaA*	0.0	0.51
FnrS : *sodA*	0.0	0.76
FnrS : *sodB*	1.6	0.34
FnrS : *metE*	0.0	0.81
FnrS : *cydD*	0.0	0.25
FnrS : *folE*	0.0	0.32
FnrS : *folX*	8.5	0.16
FnrS : *gpmA*	1.3	0.51
FnrS : *maeA*	15.4	0.56
FnrS : *yobA*	49.2	0.49
FnrS : *marA*	18.7	0.4
FnrS : *iscR*	22.1	0.81
FnrS : *nagZ*	0.0	0.19
FnrS : *sdhA*	0.0	0.53
SdsR : *mutS*	5.8	0.05
SdsR : *tolC*	0.0	0.13
SdsR : *zapG*	0.0	0.15
DicF : *ftsZ*	0.0	0.0
ArrS : *gadE*	0.0	0.95
DsrA : *hns*	12.3	0.53
DsrA : *rpoS*	254.2	0.79

Supplementary Figure 4

The figure indicates 134 experimentally confirmed sRNA:target interactions in *E. coli* [3]. For each interaction, the normalized odds ratio determined from RIL-seq experiments [4] is indicated, with RIL-seq identified interactions shown in green. Additionally, for each interaction, its predicted probability of being an interaction, as determined by our machine learning model, is indicated, with interactions predicted by our model shown in purple. RIL-seq identifies 68 of the 134 interactions. Our model identifies 107 of the 134 interactions.

GcvB Target	RIL-Seq (Odds Ratio)	Prediction (Probability)
yifK	92.8	0.96
zitB	18.9	0.3
yhjE	18.8	0.99
ydeE	15.6	0.2
ylcI	13.4	0.82
livJ	11.0	0.98
ysgA	10.9	0.92
yafT	10.9	0.75
ybhP	10.7	0.16
aroP	10.5	0.97
livK	10.1	0.97
abgB	10.1	0.2
cstA	9.9	0.8
gdhA	9.5	0.98
nlpA	9.1	0.96
dtpA	8.6	0.9
yeiG	8.4	0.91
ilvM	7.9	0.26
zapE	7.3	0.61
ydhS	7.2	0.05
sstT	7.1	0.98
glcD	6.8	0.82
brnQ	6.4	0.97
rihA	6.3	0.19
cfa	6.3	0.58
cycA	6.0	0.98
gltI	6.0	0.97
purU	5.7	0.54
ilvC	5.7	0.68
ychJ	5.3	0.5
dppA	5.2	0.97
fiu	5.1	0.65
oppA	4.9	0.93
yjiH	4.5	0.46
serA	4.3	0.82
mcrB	4.2	0.24
lldP	4.1	0.6
cysJ	4.1	0.98
ydcD	4.1	0.32
gltP	4.0	0.82
hofM	3.7	0.24
asd	3.7	0.4
trpC	3.5	0.24
ydiJ	3.5	0.86
argP	3.3	0.96
waaP	3.1	0.08
yfhM	3.1	0.15
fhuE	3.1	0.98
thrL	2.9	0.15
nanC	2.7	0.9
rppH	2.4	0.84
yddB	2.2	0.04
ybaN	2.0	0.18
yihY	2.0	0.04
yagU	2.0	0.88
dfp	1.8	0.45
yciG	1.8	0.74
yffR	1.8	0.01
sdhE	1.8	0.2
trpE	1.7	0.88
argT	1.7	0.98
trpD	1.6	0.57
dtpB	1.6	0.79
metI	1.6	0.15
aroC	1.5	0.82
maeB	1.5	0.85
kgtP	1.4	0.38
dcyD	1.4	0.94
mltC	1.3	0.64
ebgR	1.3	0.55
asnA	1.3	0.81
panD	1.2	0.5
yciA	1.2	0.11
rffM	1.2	0.37
cysB	1.1	0.56
rhlB	1.0	0.65
phoP	1.0	0.58
fabI	1.0	0.93
panB	1.0	0.46
bamD	1.0	0.4
fliK	1.0	0.34
alr	0.9	0.24
acs	0.9	0.99
ivbL	0.9	0.07
envC	0.8	0.31
ynaE	0.7	0.05
rodZ	0.7	0.67
glpC	0.7	0.12
envZ	0.6	0.14
lrp	0.6	0.98
icd	0.6	0.83
inaA	0.6	0.87
ydcL	0.6	0.8
dicA	0.5	0.11
gatY	0.5	0.7
map	0.4	0.82
glpF	0.4	0.75
ompX	0.4	0.11
rpsT	0.4	0.19
yoaB	0.4	0.35
raiA	0.4	0.79
pheL	0.4	0.01
pmrD	0.3	0.14
malE	0.3	0.82
nanK	0.3	0.1
fecA	0.3	0.51
nanA	0.3	0.26
lipA	0.3	0.21
flgN	0.3	0.05
yidF	0.3	0.09
garD	0.2	0.9
soxS	0.2	0.55
rmf	0.2	0.73
pepQ	0.2	0.72
ftsB	0.2	0.81
recA	0.2	0.21
fliL	0.2	0.34
ompA	0.1	0.65
gpmM	0.1	0.71
adk	0.1	0.29
tpx	0.1	0.2

Supplementary Figure 5

For the sRNA GcvB, RIL-seq experiments [4] suggested 121 targets. The normalized odds ratio determined from RIL-seq experiments is indicated, with RIL-seq suggested targets shown in green. For each RIL-seq suggested target, its predicted probability of being a target, as determined by our machine learning model, is indicated, with targets predicted by our model shown in purple. Our model predicts 70 of the 121 targets.

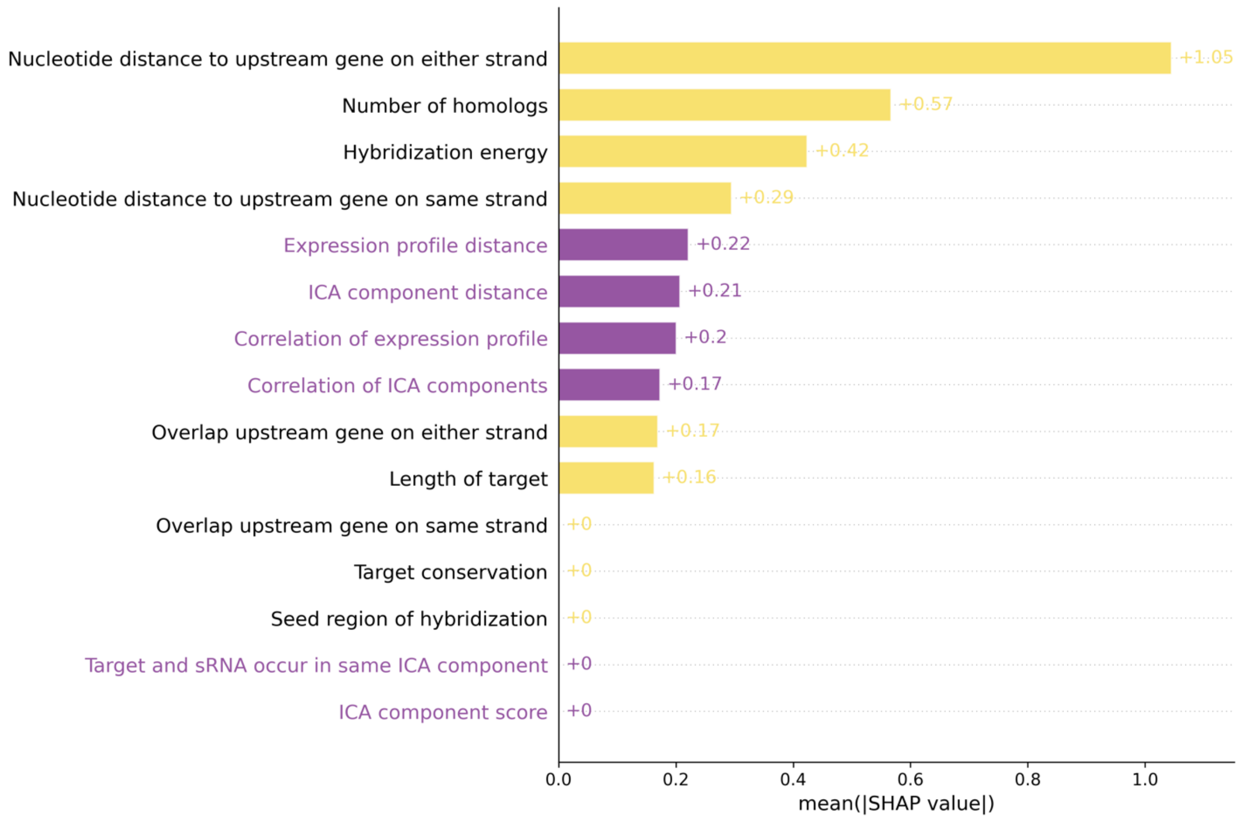

Supplementary Figure 6

For the model trained on *Salmonella* data, Shapley values are indicated for the 15 features, with the 6 new expression features indicated in purple. Larger Shapley values for a feature correspond to a greater contribution toward the model's output.

Supplementary Table 1

The table contains details about the compendia of RNA-seq experiments. There are two tabs, one for *E. coli* and one for *Salmonella*.

Supplementary Table 2

The table indicates the performance of six different machine learning algorithms on validation data for each of four datasets: the small set of *E. coli* data, the medium set of *E. coli* data, the large set of *E. coli* data, and the set of *Salmonella* data. Performance measures include sensitivity, false positive rate (FPR), and area under the receiver operating characteristic curve (AUC). The table includes six tabs, one for each algorithm investigated: gradient boosting, *k* nearest neighbors, logistic regression, random forests, support vector machines, and neural networks. For each algorithm a variety of hyperparameter values were explored using grid search. For gradient boosting, hyperparameters include the minimum number of examples required for a leaf, the number of boosting stages, the learning rate, and the maximum tree depth. For *k* nearest neighbors, hyperparameters include the number of neighbors *k*, the weight function, and the norm of the distance function. For logistic regression, hyperparameters include the inverse regularization strength, the solver algorithm used for optimization, and the norm of the penalty. For random forests, hyperparameters include the maximum tree depth, the number of trees in the forest, and the minimum number of examples required to split an internal tree node. For support vector machines, hyperparameters include the regularization strength, the kernel, and gamma the kernel coefficient. For neural networks, hyperparameters include the number of hidden layers and units per hidden layer, the activation function used in the hidden layers, the regularization strength, and the maximum number of iterations.

Supplementary Table 3

The table indicates, for each sRNA and candidate target, the machine learning algorithm's prediction as to whether the candidate is indeed a regulatory target of the sRNA or not. Both the prediction and the probability that the candidate is a target, as determined by the algorithm, are provided. The table includes two tabs, one for *E. coli* predictions and one for *Salmonella* predictions. In the *E. coli* tab, confirmed targets as part of the small dataset [3] are indicated as well as normalized odds ratios based on RIL-seq experiments [4]. In the *Salmonella* tab, confirmed targets [3] are indicated.

References

1. Salgado, H., et al., *RegulonDB v12.0: a comprehensive resource of transcriptional regulation in E. coli K-12.* Nucleic Acids Res, 2024. **52**(D1): p. D255-D264. PMC10767902
2. Karp, P.D., et al., *The EcoCyc Database (2023).* EcoSal Plus, 2023. **11**(1): p. eesp00022023. PMC10729931
3. Hor, J., et al., *Trans-Acting Small RNAs and Their Effects on Gene Expression in Escherichia coli and Salmonella enterica.* EcoSal Plus, 2020. **9**(1). PMC7112153
4. Melamed, S., et al., *Global Mapping of Small RNA-Target Interactions in Bacteria.* Mol Cell, 2016. **63**(5): p. 884-97. PMC5145812
